# Deciphering the Benefits and Risks of Single-Inhaler Triple Therapy in COPD

**DOI:** 10.3390/arm94050070

**Published:** 2026-09-30

**Authors:** Ivan Kopitovic, Sanja Dimic-Janjic, Mihailo Stjepanovic, Sanja Hromis, Biljana Zvezdin, Miroslav P. Ilic, Violeta Kolarov, Rade Milic, Ivan Cekerevac, Vojislav Cupurdija, Ivana Buha, Jelena Jankovic, Maja Omcikus, Nikola Trboljevac, Ivana Stankovic, Marija Vukoja

**Affiliations:** 1 Faculty of Medicine, University of Novi Sad, 21000 Novi Sad, Serbia; shromis@gmail.com (S.H.); biljanazvezdin@yahoo.com (B.Z.); micailic@yahoo.com (M.P.I.); violeta.kolarov@gmail.com (V.K.); 2 Institute for Pulmonary Diseases of Vojvodina, 21204 Sremska Kamenica, Serbia; 3 Faculty of Medicine, University of Belgrade, 11000 Belgrade, Serbia; sanjadimicjanjic@gmail.com (S.D.-J.); mihailostjepanovic@gmail.com (M.S.); ivanabuha33@gmail.com (I.B.); jjelena1984@gmail.com (J.J.); 4 Clinic for Pulmonology, University Clinical Center of Serbia, 11000 Belgrade, Serbia; nikola.trboljevac@gmail.com; 5 Medical Faculty of the Military Medical Academy, University of Defense in Belgrade, 11000 Belgrade, Serbia; rade.milic1975@gmail.com; 6 Clinic for Pulmonology, Military Medical Academy, 11000 Belgrade, Serbia; 7 Faculty of Medicine, University of Kragujevac, 34000 Kragujevac, Serbia; icekerevac@gmail.com (I.C.); vojacup@gmail.com (V.C.); 8 Clinic for Pulmonology, University Clinical Center of Kragujevac, 34000 Kragujevac, Serbia; 9 Faculty of Medicine, University of Nis, 18000 Nis, Serbia; staivana@gmail.com; 10 Clinic for Pulmonology, University Clinical Center of Nis, 18000 Nis, Serbia

**Keywords:** chronic obstructive pulmonary disease, single-inhaler triple therapy, inhaled corticosteroids, exacerbations, mortality, cardiovascular safety, blood eosinophils, target trial emulation, adherence, precision prescribing

## Abstract

**Highlights:**

**What are the main findings?**

- Triple pharmacological therapy reduces moderate-to-severe COPD exacerbations in appropriately selected patients. When the same three drug classes are prescribed, a single inhaler may simplify treatment compared with multiple inhalers.
- The benefit–risk balance is shaped by exacerbation history, blood eosinophil count, pneumonia susceptibility, cardiovascular comorbidity, previous corticosteroid exposure, and the patient’s ability to use the prescribed inhaler consistently.

**What are the implications of the main findings?**

- Treatment should be individualized, considering each patient’s exacerbation frequency, blood eosinophil count, pneumonia and cardiovascular risk, previous response to inhaled corticosteroids, inhaler technique, and expected adherence.
- Mortality and cardiovascular findings depend on the comparator, study design, and treatment received before randomization; carefully designed target trial emulations may clarify whether these associations are causal.

**Abstract:**

Single-inhaler triple therapy (SITT) combines an inhaled corticosteroid (ICS; for example, fluticasone furoate, budesonide, or beclometasone dipropionate), a long-acting β2-agonist (LABA; for example, vilanterol or formoterol fumarate), and a long-acting muscarinic antagonist (LAMA; for example, umeclidinium or glycopyrronium bromide) in one device. Triple pharmacological therapy reduces exacerbations in selected patients with chronic obstructive pulmonary disease (COPD), but its effects on mortality and cardiovascular outcomes remain uncertain. This structured narrative review summarizes randomized trials, observational comparative-effectiveness studies, target trial emulations, systematic reviews, and health technology assessments published between 2015 and 2026. We grouped the evidence by comparator: LABA/LAMA, ICS/LABA, multiple-inhaler triple therapy (MITT), and ICS continuation versus withdrawal, because each comparison addresses a different clinical question. Pivotal trials and evidence syntheses consistently showed fewer moderate-to-severe exacerbations with triple therapy, with reported rate ratios of approximately 0.69–0.93. Mortality signals from IMPACT and ETHOS are clinically important, but previous ICS use and ICS withdrawal among participants assigned to ICS-free comparators make these findings difficult to interpret. Observational cardiovascular findings are also uncertain because residual confounding, treatment selection, exposure misclassification, outcome misclassification, and differences in baseline disease severity limit causal interpretation. Compared with MITT, the main advantages of SITT are simpler treatment and possible improvements in adherence and persistence, not greater pharmacological potency. Treatment decisions should consider exacerbation history, repeated blood eosinophil counts, pneumonia risk, cardiovascular disease, previous ICS exposure and response, inhaler technique, device suitability, and patient preference. Trials in patients with documented absence of previous ICS use and well-designed target trial emulations are needed to determine whether the observed mortality and cardiovascular associations are causal.

## 1. Introduction

Chronic obstructive pulmonary disease (COPD) causes substantial illness and death. Patients have persistent respiratory symptoms, airflow limitation, recurrent exacerbations, and common systemic comorbidities [1,2]. Diagnosis and treatment are often delayed, which may reduce opportunities to improve symptoms and prevent exacerbations [3]. Long-acting β2-agonists (LABAs) and long-acting muscarinic antagonists (LAMAs) are the main maintenance bronchodilators. Inhaled corticosteroids (ICSs) are added mainly for patients who remain at risk of exacerbations and are likely to respond to corticosteroids [4,5,6,7]. Current GOLD recommendations consider exacerbation history, symptoms, blood eosinophil count (BEC), previous treatment, comorbidities, inhaler technique, and patient preference rather than spirometric severity alone [1,2,5,6,7]. Earlier identification of COPD with mild airflow limitation may provide opportunities for smoking cessation, reduction in modifiable risk factors, closer clinical monitoring, and timely treatment, although evidence that early pharmacological intervention modifies long-term disease progression remains limited [8].

Triple pharmacological therapy combines ICS, LABA, and LAMA [9]. Single-inhaler triple therapy (SITT) delivers these classes through one device, whereas multiple-inhaler triple therapy (MITT) uses two or more devices. Triple therapy is not the initial treatment for most patients. GOLD 2026 recommends LABA/LAMA as a preferred initial maintenance regimen for many symptomatic patients [1,2]. Trials of dual bronchodilation [10] and LAMA monotherapy [11] provide context for these options. Escalation to triple therapy is intended for patients who continue to exacerbate despite LAMA/LABA, particularly when blood eosinophil counts are elevated; persistent dyspnoea alone is not an indication to add ICS [5]. Randomized trials underpin these pathways, while target trial emulation provides a framework for assessing treatment strategies using observational data [12,13]. Within the RCT-DUPLICATE initiative, an observational database emulation of IMPACT produced an exacerbation estimate for triple therapy versus dual bronchodilation that differed from the randomized-trial estimate. The investigators identified trial-mandated discontinuation of previous treatment, including ICS among many IMPACT participants, as an important design feature that could not be reproduced fully in routine-care data [14]. The single-inhaler triple regimens evaluated in the pivotal trials are fluticasone furoate/umeclidinium/vilanterol (FF/UMEC/VI) [15], budesonide/glycopyrronium/formoterol (BUD/GLY/FOR) [16,17], and extrafine beclometasone dipropionate/formoterol/glycopyrronium (BDP/FF/GB) [18,19,20].

Evidence syntheses have evaluated single-inhaler triple therapy against dual therapy or separate triple therapy [21]. A retrospective study assessed outcomes after switching from multiple inhalers to SITT [22]. Other meta-analyses evaluated triple therapy against single and dual long-acting therapies [23,24]. A pooled real-world study examined effectiveness, safety, and adherence with extrafine BDP/FF/GB [25]. This review adds a critical analysis based on the comparator and the patient’s previous treatment. It separates three questions: adding or continuing ICS, adding a second long-acting bronchodilator, and changing established triple therapy from several inhalers to one inhaler. It also reviews recent evidence on previous ICS exposure and withdrawal, cardiovascular outcomes, adherence, device burden, target trial emulation, and health-system value. This approach helps explain why mortality estimates differ across LABA/LAMA, ICS/LABA, and MITT comparators and highlights the limited evidence in patients with documented absence of previous ICS use.

## 2. Materials and Methods

We conducted a structured narrative review of MEDLINE, EMBASE, Cochrane CENTRAL, and ClinicalTrials.gov for records published from 1 January 2015 to 31 July 2026. Search terms covered COPD, single-inhaler and multiple-inhaler triple therapy, ICS withdrawal, exacerbations, mortality, cardiovascular outcomes, pneumonia, blood eosinophils, adherence, persistence, cost-effectiveness, and target trial emulation. The search was adapted for each database. We also checked the reference lists of key trials, reviews, guidelines, and health technology assessments. Important studies published before 2015 were included when they were needed to interpret current evidence.

Eligible studies included adults with spirometrically confirmed COPD and examined SITT, triple pharmacological therapy, ICS continuation or withdrawal, adherence, safety, cardiovascular or mortality outcomes, or economic outcomes. We included randomized trials, systematic reviews and meta-analyses, comparative observational studies, target trial emulations, and health technology assessments. We excluded asthma-only studies without separate COPD data, non-comparative opinion articles, case reports, paediatric studies, and reports that did not address one of the clinical questions defined for this narrative synthesis. General methodological articles were used to inform study-design interpretation but were not treated as COPD clinical-effectiveness evidence.

The review team first screened titles and abstracts and then assessed potentially relevant full texts. Uncertainties about eligibility were resolved through team discussion. Because this was a structured narrative review, we did not use formal duplicate systematic-review screening or prepare a PRISMA flow diagram. We grouped evidence by clinical question and comparator: triple therapy versus LABA/LAMA, triple therapy versus ICS/LABA, SITT versus MITT, and ICS continuation versus withdrawal. We gave particular attention to studies that aligned eligibility, treatment assignment, start of follow-up, outcome definitions, and analysis according to target trial principles [12,13].

Risk of bias was assessed qualitatively to guide interpretation rather than to produce one numerical quality score. Randomized trials were considered using RoB 2 domains. Observational studies were appraised qualitatively with reference to Newcastle–Ottawa domains and design-specific sources of bias, together with attention to new-user design, active comparators, confounding by indication, treatment selection, exposure and outcome misclassification, and time-related bias. We did not perform formal duplicate scoring or exclude studies according to a numerical threshold. We did not pool results because the studies differed substantially in design, comparator, population, outcome definitions, and follow-up. The review was not registered in PROSPERO because it was designed as a structured narrative synthesis rather than a systematic review. The findings should therefore be interpreted as a critical integration of heterogeneous evidence, not as an exhaustive or formally pooled treatment-effect estimate.

## 3. Results

### 3.1. Clinical Efficacy and Patient-Centred Outcomes

#### 3.1.1. Exacerbation Reduction and Lung Function

The clearest benefit of triple pharmacological therapy is fewer moderate-to-severe exacerbations. In IMPACT, FF/UMEC/VI reduced exacerbations compared with both ICS/LABA and LAMA/LABA [15]. ETHOS also showed fewer exacerbations with BUD/GLY/FOR, although the effect differed by corticosteroid dose [16]. KRONOS mainly showed improved lung function over 24 weeks and did not require a previous exacerbation [17]. TRIBUTE reported an approximately 15% reduction in moderate-to-severe exacerbations with extrafine BDP/FF/GB compared with LAMA/LABA [18]. TRILOGY and TRINITY provided earlier evidence for extrafine triple therapy [19,20]. Meta-analyses report fewer exacerbations and, in some comparisons, modest improvements in lung function and health status [21,23,24]. The magnitude of benefit depends on the population and comparator [21,23,24].

Triple pharmacological therapy must be distinguished from its method of delivery. SITT and MITT contain the same three drug classes. Therefore, differences between them are more likely to result from dosing frequency, the number of inhalers, adherence, persistence, or inhaler technique than from stronger pharmacological activity. RESTART and other real-world switching studies reported improvements after patients changed to one inhaler, but their non-randomized designs limit causal conclusions [22,26]. INTREPID found a 50 mL greater improvement in FEV_1_ and better health-status outcomes with once-daily FF/UMEC/VI than with non-ELLIPTA MITT, while critical inhaler-error and pneumonia rates were similar [27]. However, INTREPID was open-label, which may have affected spirometry and other assessments. Overall, SITT is an effective and simpler way to deliver indicated triple therapy, but using one device should not be interpreted as greater pharmacological potency.

#### 3.1.2. Comparator-Specific Mortality and Cardiovascular Evidence

Whether triple therapy directly lowers mortality beyond its effect on exacerbations remains unresolved. The interpretation depends on the comparator and previous ICS exposure.

Earlier evidence from TORCH suggested a possible survival effect of ICS/LABA therapy compared with placebo, but the prespecified mortality analysis did not reach conventional statistical significance [28].

Compared with LABA/LAMA, IMPACT reported lower all-cause mortality with FF/UMEC/VI (HR 0.72, 95% CI 0.53–0.99) [29], and ETHOS reported a similar signal for the higher budesonide dose [16]. These findings are clinically important, but neither trial was designed with mortality as the prespecified primary endpoint for which the trial was statistically powered; the number of deaths was small, and more than 70% of IMPACT participants and approximately 80% of ETHOS participants were receiving ICS before randomization [30,31]. Participants assigned to LABA/LAMA could therefore experience abrupt ICS withdrawal. A 2026 re-analysis reported that, in ETHOS, previous ICS users assigned to LABA/LAMA had higher mortality than previous ICS users assigned to ICS-containing triple therapy, whereas no significant mortality difference was observed among previous ICS non-users [32]. Published IMPACT strata showed a similar pattern [32]. This analysis supports a withdrawal explanation but does not prove that withdrawal accounts for the entire mortality difference. Trial investigators have also argued that IMPACT was not designed as an ICS-withdrawal study and that the distribution and timing of deaths do not demonstrate a withdrawal surge. The mortality signal should therefore be treated as uncertain rather than as proof of either benefit or harm [30,31,32,33].

As context for choosing a dual regimen, FLAME compared LABA/LAMA with ICS/LABA and found fewer moderate-to-severe exacerbations with dual bronchodilation, without a significant mortality difference [34]. This was not a trial of triple therapy. For the distinct triple-therapy versus ICS/LABA comparison, evidence does not establish a consistent survival advantage. In ETHOS, higher-dose budesonide/glycopyrronium/formoterol did not significantly reduce mortality versus budesonide/formoterol [16]. Observational comparisons address different treatment contrasts: SITT versus ICS/LABA [35], triple therapy versus dual bronchodilation [36], and SITT versus MITT [37]. Their estimates should not be combined as evidence for a single pharmacological step-up. A 2025 meta-analysis likewise found no significant mortality advantage for single-inhaler triple therapy versus ICS/LABA [38]. Because both groups continue ICS, this comparison is less susceptible to corticosteroid-withdrawal effects than comparisons against LABA/LAMA and primarily evaluates the incremental effect of adding a LAMA to an ICS/LABA background. Collectively, these findings support the view that ICS-containing regimens should be selectively prescribed for appropriate patients rather than used routinely.

Compared with MITT, both groups receive the same three pharmacological classes; this comparison mainly tests delivery through one inhaler. SKOPOS-MAZI reported lower mortality (HR 0.82, 95% CI 0.75–0.91) and a lower risk of a first severe cardiopulmonary event (HR 0.88, 95% CI 0.83–0.93) with BUD/GLY/FOR SITT versus MITT [37]. These findings may reflect better adherence or continuity, but residual confounding, exposure misclassification, and outcome misclassification preclude a causal survival claim.

Cardiovascular evidence is also inconsistent. A 2025 active-comparator analysis reported more major adverse cardiovascular events with SITT than with ICS/LABA [35], whereas SKOPOS-MAZI favoured SITT over MITT [37]. These estimates address different treatment decisions and arise from observational data. They remain vulnerable to treatment selection, incomplete measurement of COPD severity and cardiovascular risk, and residual confounding. Randomized evidence offers better control of baseline confounding but includes selected populations, few cardiovascular events, and endpoints that were not consistently prospectively powered. Cardiovascular safety therefore remains unresolved and should be interpreted by comparator, previous treatment, and study design.

### 3.2. Safety Profile: Balancing ICS Benefit and Pneumonia Risk

In practice, the decision to use an ICS-containing regimen depends mainly on balancing exacerbation prevention against pneumonia risk. BEC modifies the expected preventive response to ICS. Pneumonia susceptibility is also influenced by age, previous pneumonia, low body mass, smoking, airflow limitation, bronchiectasis, chronic airway infection, corticosteroid molecule and dose, and other comorbidities. Lower eosinophil counts often indicate less expected ICS benefit and, in some cohorts, greater infection susceptibility. This is a clinical benefit–risk relationship, not a contradiction requiring resolution.

#### 3.2.1. ICS-Related Pneumonia Risk

Pneumonia is the principal recognized harm associated with ICS-containing COPD treatment. Local immunosuppression, impaired innate host defence, and changes in the airway microbiome are plausible mechanisms [39,40]. In the Cochrane analysis by Kew and Seniukovich, fluticasone was associated with an increase in non-fatal pneumonia (odds ratio 1.78, 95% CI 1.50–2.12), while the estimate for budesonide was smaller and less precise (odds ratio 1.62, 95% CI 1.00–2.62); neither drug showed a significant effect on overall mortality [39]. Cross-trial comparisons between molecules should nevertheless be cautious because dose, device, population, pneumonia definition, and follow-up differ. No head-to-head pneumonia trial comparing fluticasone furoate and budesonide in the same SITT formulation exists; the Cochrane signal therefore reflects different trials, populations, and definitions. For clinical decisions, previous pneumonia, recurrent lower respiratory infection, bronchiectasis, low body mass, severe airflow limitation, ongoing smoking, and limited exacerbation history should be considered alongside BEC. The relevant question is not whether ICS causes pneumonia in every patient, but whether the anticipated exacerbation reduction justifies the incremental infection risk for the individual patient.

#### 3.2.2. Blood Eosinophil Thresholds: The GOLD Framework

Blood eosinophil count is a continuous, probabilistic biomarker of the exacerbation-prevention response to ICS rather than a binary treatment mandate [41]. In WISDOM, the risk associated with ICS withdrawal increased progressively at higher eosinophil counts, with the clearest excess among patients near or above 300 cells/µL or 4–5% [42]. Other cohorts and exacerbation studies support eosinophils as a marker of corticosteroid responsiveness, although percentages and absolute counts are not directly interchangeable, and acute illness or systemic corticosteroid use can alter measurements [43,44]. Longitudinal analyses also show substantial within-patient variability, so repeated stable-state measurements are preferable when the treatment decision is uncertain [45]. Consistent with GOLD, three ranges are clinically useful, but they should be interpreted in conjunction with exacerbation history rather than as absolute rules:

- **<100 cells/µL**: little or no expected preventive benefit from adding ICS for exacerbations in most patients; consider an ICS-sparing regimen unless another clear indication exists.
- **100–299 cells/µL**: the expected benefit increases across the range; individualize treatment according to the number and severity of exacerbations, previous ICS response, pneumonia risk, and comorbidities.
- **≥300 cells/µL**: a greater probability of benefit from ICS, particularly in patients with recurrent or severe exacerbations; avoid abrupt withdrawal without a clear indication and a monitored plan.

#### 3.2.3. ICS De-Escalation

De-escalation should be considered when the original indication for ICS is weak, adverse effects occur, pneumonia risk is high, or triple therapy has continued without demonstrable benefit. The decision should distinguish patients receiving ICS without a clear indication from those with recurrent exacerbations and an eosinophilic phenotype. A population-based study comparing LABA/ICS with LAMA as initial treatment found that the relative effectiveness of ICS-containing treatment varied with exacerbation history and blood eosinophil count [46]. BEACON supports the bronchodilator efficacy of indacaterol/glycopyrronium and provides a pharmacological basis for ICS-sparing treatment [47]. In SUNSET, withdrawal to indacaterol/glycopyrronium increased exacerbation risk mainly among patients with BEC ≥ 300 cells/µL [48]. WISDOM found that gradual withdrawal did not increase moderate-to-severe exacerbations in the overall study population, but risk rose with increasing eosinophil count [42,49]. Conversely, discontinuing ICS was associated with a 37% reduction in serious pneumonia in a large observational cohort, with a larger reduction after fluticasone than budesonide [50]. A safe step-down strategy therefore requires confirmation of the original indication, repeated BEC where feasible, review of exacerbations and pneumonia, optimization of dual bronchodilation, gradual rather than abrupt withdrawal when clinically appropriate, and early follow-up after the change.

### 3.3. Real-World Effectiveness: Adherence, Persistence, and TTE

SITT may improve real-world effectiveness by reducing the number of devices, dosing occasions, and technique requirements. Italian database evidence suggests fewer exacerbations and lower rescue or systemic corticosteroid use among persistent users of BUD/GLY/FOR, although pre-post and persistent-user designs remain vulnerable to selection and regression-to-the-mean effects [51]. When comparing SITT regimens, a US claims analysis found higher adherence and persistence with once-daily than with twice-daily dosing [52]. A conference-abstract narrative review [53], along with studies from Spain and Japan [54,55], reported higher adherence and persistence with SITT than with MITT. These findings are plausible because adherence to inhaled therapy is often poor and tends to decline as regimen complexity increases [56,57]. Multiple devices may require different inspiratory manoeuvres and create more opportunities for critical errors, although a single device does not remove the need for technique assessment and repeated instruction [58,59]. Lower dosing frequency has also been associated with better adherence and lower healthcare utilization [60]. Nevertheless, dispensing records do not confirm correct inhalation or actual drug exposure, and patients switched to SITT may differ systematically from those who remain on MITT. The most defensible interpretation is therefore that SITT can facilitate delivery when triple therapy is already indicated; simplification alone does not justify escalation. Exacerbation risk, BEC, previous treatment response, device suitability, and patient preference should remain central to prescribing [61,62]. Target trial emulation can strengthen comparative observational research by specifying eligibility criteria, treatment strategies, treatment assignment, time zero, follow-up, outcomes, and the causal contrast before analysis [12,13]. It cannot, however, reproduce every feature of a randomized trial. In the RCT-DUPLICATE initiative, the observational emulation of IMPACT produced an exacerbation estimate that differed from the randomized result. The investigators attributed this discordance in part to trial-mandated discontinuation of previous treatment, which could not be replicated fully among routine-care treatment initiators [14]. This example reinforces the need to distinguish treatment initiation from treatment continuation or withdrawal when interpreting observational comparisons of triple therapy.

### 3.4. Health-System Implementation and Global Pharmacoeconomics

Implementation and economic value vary substantially across healthcare systems. Prescribing is influenced not only by clinical phenotype but also by specialist access, reimbursement rules, formulary restrictions, clinician preference, and continuity after hospital discharge [62]. In Italy, AIFA Nota 99 has shaped who may initiate or continue fixed-dose triple therapy, demonstrating how policy can affect uptake independently of clinical evidence [63]. Canadian public plans likewise apply jurisdiction-specific listing and reimbursement criteria [64]. Economic models from the United Kingdom and Germany generally found SITT cost-effective relative to multiple-inhaler strategies when reductions in exacerbations and hospital use offset higher acquisition costs [65,66]. A systematic review reached a similar conclusion for many high-income settings, particularly among severe, exacerbation-prone populations [67], and IMPACT-based modelling supported lifetime value under assumptions of sustained treatment effects [68]. The economic conclusions depend on model assumptions about drug prices, exacerbations, hospital use, adherence, and time horizon [65,66,67,68]. UK observational cost data separately show that management costs vary with exacerbation frequency [69]. In Thailand, current prices produced incremental cost-effectiveness ratios above the national willingness-to-pay threshold, and a substantial price reduction was required for favourable value estimates [70]. Serbian evidence also highlights variation in post-discharge optimization and implementation pathways, which may limit realized effectiveness even when a therapy is clinically appropriate [71]. Local, independent health technology assessment is therefore preferable to transferring cost-effectiveness conclusions across jurisdictions. The principal COPD triple-therapy studies and their key methodological considerations are summarized in Table 1.

## 4. Discussion

This review separates established benefits from uncertain effects. Randomized trials and evidence syntheses consistently support fewer moderate-to-severe exacerbations with triple pharmacological therapy. In contrast, mortality and cardiovascular estimates depend on comparator choice, previous ICS exposure, treatment withdrawal, study design, and baseline risk [15,16,29,30,31,32,33,35,36,37,38]. The term “triple-therapy benefit” may describe three different effects: adding ICS to LABA/LAMA, adding a LAMA to ICS/LABA, or consolidating established triple therapy into one inhaler. These effects should not be treated as interchangeable.

The review extends previous syntheses by placing these three causal questions in one framework and by integrating recent withdrawal re-analyses, target trial principles, real-world delivery outcomes, and evidence from populations with and without previous ICS use. This comparator-aware approach explains why apparently conflicting mortality and cardiovascular estimates can coexist. It also prevents benefits attributable to continued ICS or improved adherence from being attributed automatically to the single-inhaler device itself.

Four clinical implications follow from this evidence.

First, the IMPACT and ETHOS mortality findings should be regarded as hypothesis-strengthening rather than definitive proof of a direct survival effect [15,16,29,30,31]. Prior ICS use was common, mortality was not the prespecified primary endpoint for which the trial was statistically powered, and assignment to an ICS-free comparator may have caused abrupt withdrawal in susceptible participants [32,33]. The 2026 re-analysis found mortality patterns consistent with withdrawal-related harm, particularly among previous ICS users, but this was a secondary analysis of published trial data and cannot determine how much of the observed difference was caused by withdrawal [32]. The safest conclusion is that inappropriate ICS withdrawal may harm selected patients and that current trials do not conclusively establish an independent mortality benefit from triple therapy.

Second, evidence for starting triple therapy in patients with a documented absence of previous ICS use remains limited. In the subgroup that was not using ICS before randomization, the recent ETHOS re-analysis did not identify a significant mortality benefit from starting ICS-containing triple therapy, but the estimate was imprecise [32]. Previous ICS non-use at a recorded time point should not be interpreted automatically as documented absence of prior ICS exposure. This subgroup is nevertheless important because it is less exposed to withdrawal bias and therefore addresses treatment initiation more directly. Dedicated randomized trials in exacerbation-prone patients with documented absence of previous ICS use are needed before a mortality benefit from initiating triple therapy can be claimed.

Third, cardiovascular safety remains uncertain. Active-comparator observational studies may include patients with more multimorbidity than those enrolled in pivotal trials, but these studies remain susceptible to residual confounding, treatment selection, exposure misclassification, and incomplete measurement of COPD severity and baseline cardiovascular risk [35,36,37]. Randomized trials provide stronger protection against baseline confounding but include selected populations, relatively few events, and cardiovascular endpoints that were not always prospectively powered [15,16,17,18,19,20]. Observational estimates should therefore be considered alongside, rather than given automatic precedence over, randomized evidence. Baseline cardiovascular assessment and follow-up remain prudent when treatment is changed.

Fourth, precision prescribing should extend, rather than replace, existing GOLD recommendations. GOLD identifies exacerbation history and BEC as central determinants of ICS use. The framework proposed here adds four explicit checks before prescribing or changing SITT: (1) define the treatment contrast—ICS initiation, ICS continuation or withdrawal, addition of a second bronchodilator, or device consolidation; (2) document previous ICS exposure and response; (3) balance pneumonia and cardiovascular susceptibility against expected exacerbation benefit; and (4) confirm that the selected device matches inspiratory flow, dexterity, cognition, technique, adherence barriers, and patient preference. Thus, the framework links guideline eligibility to the actual causal question and delivery context rather than proposing a new treatment algorithm.

Economic evidence reinforces the need for local decision-making. A therapy that is cost-effective where hospitalizations are expensive and drug prices are negotiated may not represent good value in a middle-income system with different thresholds, reimbursement, and access [65,66,67,68,69,70,71]. Moreover, modelled value is realized only if eligible patients receive treatment, use the device correctly, and maintain therapy. Independent analyses using local prices, event rates, and implementation costs are particularly important where reimbursement decisions may affect equity of access.

The most appropriate candidates for SITT are patients in whom triple therapy is clinically indicated and one device is suitable. This generally includes patients with continuing exacerbations despite optimized long-acting bronchodilation, especially when BEC suggests a greater probability of ICS response or previous treatment has shown benefit [1,2,39,40,41,42,43,44,45,46,47,48,49,50]. Caution is warranted with persistently low BEC, recurrent pneumonia, chronic airway infection, bronchiectasis, or little exacerbation history. For patients already stable on triple therapy, continuation or de-escalation should depend on the original indication, prior response, adverse effects, and withdrawal risk rather than on a universal step-down rule. When triple therapy is already indicated, one inhaler may reduce regimen complexity and improve adherence or persistence [22,26,27,52,53,54,55,56,57,58,59,60]. These delivery advantages do not demonstrate pharmacological superiority over MITT and do not justify ICS exposure in patients unlikely to benefit.

**Figure 1:**
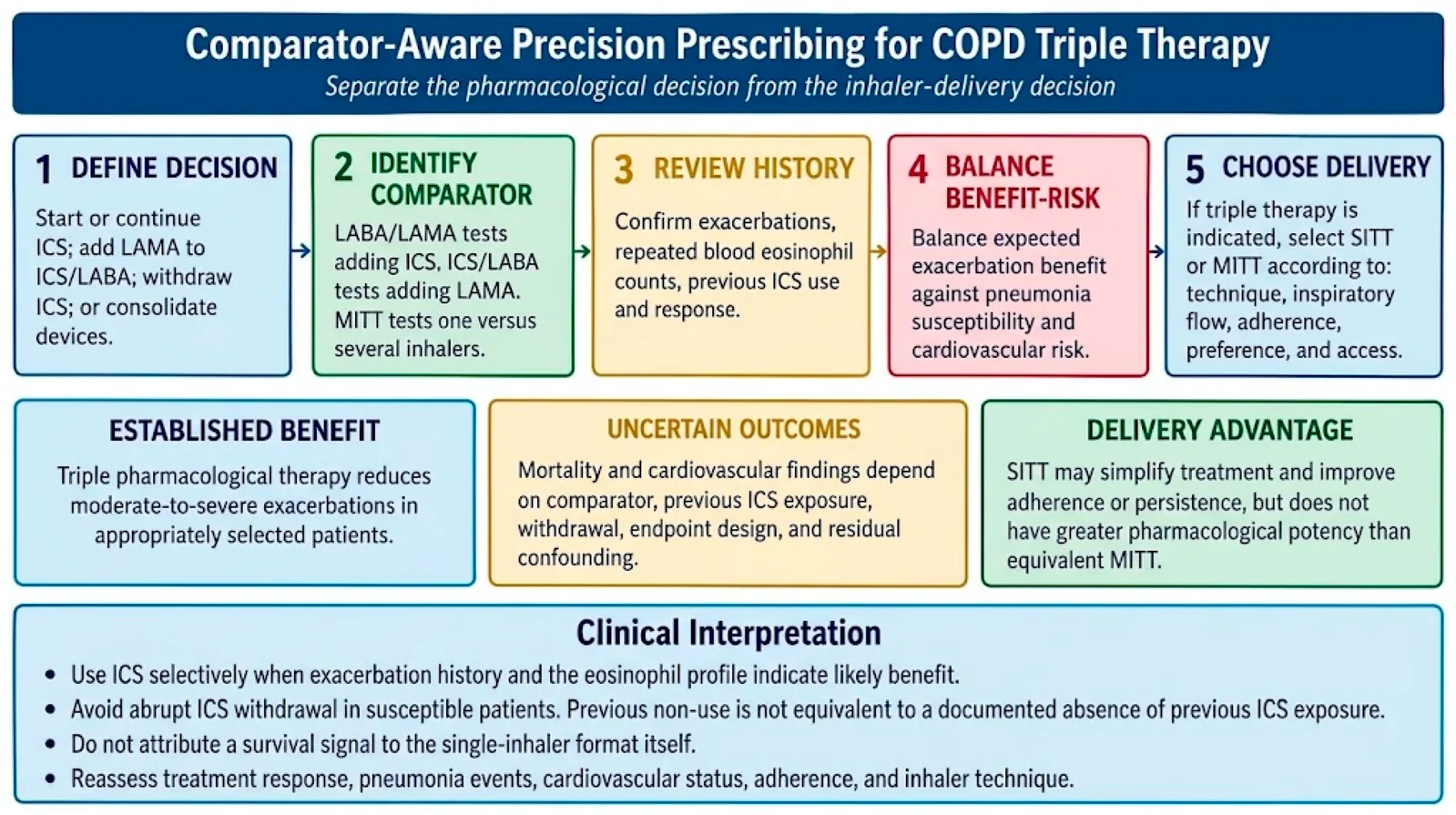
Comparator-aware precision-prescribing framework for triple therapy in COPD. The framework separates the pharmacological decision (ICS initiation, continuation, or withdrawal; addition of a second bronchodilator) from the inhaler-delivery decision (SITT versus MITT), then integrates exacerbation history, repeated BEC, previous ICS exposure and response, pneumonia and cardiovascular risk, inhaler technique, device suitability, adherence, preference, and access. Abbreviations: BEC, blood eosinophil count; COPD, chronic obstructive pulmonary disease; ICS, inhaled corticosteroid; LABA, long-acting β_2_-agonist; LAMA, long-acting muscarinic antagonist; MITT, multiple-inhaler triple therapy; SITT, single-inhaler triple therapy.

Figure 1 summarizes this comparator-aware extension of guideline-based prescribing. It first identifies the decision being evaluated: starting or continuing ICS, adding a second bronchodilator, withdrawing ICS, or consolidating devices. It then integrates exacerbation history and repeated BEC with pneumonia and cardiovascular risk, previous ICS response, adherence, inhaler technique, device suitability, patient preference, and local access. Randomized trials provide the strongest evidence for exacerbation efficacy. Well-designed observational studies can extend evidence on safety and effectiveness, while target trial principles strengthen causal interpretation by aligning eligibility criteria, treatment strategies, treatment assignment, time zero, follow-up, outcomes, and analysis [12,13]. The RCT-DUPLICATE experience also shows that observational emulation may remain discordant with the original trial when trial-mandated pretreatment changes cannot be reproduced in routine-care data [14]. These considerations are particularly important when interpreting COPD mortality and cardiovascular studies with different comparators and previous-treatment patterns [32,33,34,35,36,37,38].

## 5. Limitations

- Review design: As a structured narrative review, study identification and selection were not governed by a registered systematic-review protocol. Screening was not performed as a formal duplicate process, and publication selection may have been influenced by author judgment.
- Heterogeneity: Populations, treatment histories, comparators, outcome definitions, and follow-up differed substantially, precluding a meaningful pooled estimate across the complete evidence base.
- Mortality and cardiovascular uncertainty: Pivotal trials assessed these outcomes but did not designate them as prespecified primary endpoints and were not powered accordingly. Observational analyses remain vulnerable to residual confounding, treatment selection, exposure misclassification, and outcome misclassification.
- Generalizability: Evidence is limited for patients with documented absence of previous ICS use, very old adults, patients with substantial multimorbidity, and middle-income health systems. Pharmacoeconomic findings should not be transferred uncritically between jurisdictions.

## 6. Future Research Priorities

The following research questions are priorities:

- Patients with documented absence of previous ICS use: Compare triple therapy directly with LAMA/LABA in patients who have frequent or severe exacerbations and have no documented ICS use during a clearly defined pre-enrolment period. The studies should define mortality, pneumonia, and cardiovascular outcomes before recruitment begins.
- Comparator-specific real-world studies: Examine starting, continuing, or stopping ICS and changing from several inhalers to one inhaler as separate questions. For each question, researchers should clearly define who is eligible, which treatments are compared, when follow-up starts, and which outcomes are measured.
- Earlier triple therapy guided by blood eosinophils: Study whether patients who are at high risk of severe exacerbations and repeatedly have high blood eosinophil counts benefit from starting triple therapy earlier than current practice.
- More accurate measurement of treatment use: Combine pharmacy records with electronic inhaler monitoring, assessment of inhaler technique, and patient-reported barriers to treatment.
- Studies of pneumonia risk: Examine how the type and dose of ICS, airway bacteria, bronchiectasis, and chronic infection affect the risk of pneumonia.
- Independent economic and implementation studies: Use local drug prices, reimbursement rules, healthcare costs, and measures of fair access, especially in middle-income health systems.

## 7. Conclusions

Triple pharmacological therapy reduces exacerbations when it is clinically indicated [15,16,17,18,19,20,21,23,24]. SITT delivers the same three drug classes through a single inhaler. Its principal advantage over MITT is treatment simplification, which may improve adherence and persistence [22,26,27,53,54,55]. However, current evidence does not demonstrate that the single-inhaler format itself improves survival [22,26,27,37]. Mortality outcomes in pivotal trials were assessed, but mortality was not a prespecified primary endpoint, and the trials were not statistically powered for it; furthermore, the observed mortality signals may have been influenced by previous ICS use and ICS withdrawal in comparator groups [15,16,29,30,31,32,33]. Cardiovascular findings remain uncertain because randomized and observational studies have important but different limitations [31,35,36,37,38].

Treatment should be individualized and interpreted in relation to the comparator. Clinicians should consider exacerbation history, repeated BEC measurements, previous response to ICS, pneumonia risk, cardiovascular disease, inhaler technique, device suitability, adherence, patient preference, and treatment access [5,39,40,41,43,44,45,58,59,60,61,62]. ICS is less likely to provide benefit when exacerbation risk and BEC are low [5,42,43,44]. Conversely, abrupt ICS withdrawal may be harmful in some patients with frequent exacerbations and higher eosinophil counts [7,42,48,49]. Evidence remains insufficient to conclude that initiating triple therapy reduces mortality in patients with documented absence of previous ICS use [31,32]. Future randomized trials and carefully designed target trial emulations should distinguish the direct treatment effect from the effects of ICS withdrawal, adherence, and treatment selection [12,13,14,32,33]. Until stronger evidence becomes available, SITT should be used selectively when triple pharmacological therapy is indicated, rather than as a routine escalation strategy for all patients.

## Figures and Tables

**Table 1:** Primary COPD triple-therapy evidence and methodological interpretation. Mortality assessment or reporting does not mean that a study was prospectively designed or statistically powered to detect a mortality difference.

Study/Source	Design and Follow-Up	Primary Endpoint	Mortality Status	Powered for Mortality?	Previous ICS Exposure	Main Limitation
IMPACT [15]	RCT; 52 weeks	Moderate-to-severe exacerbation rate	Prespecified other efficacy outcome	No; sample size was based on the primary efficacy endpoint	>70%	Mortality interpretation may be affected by previous ICS use and withdrawal.
ETHOS [16]	RCT; 52 weeks	Moderate-to-severe exacerbation rate	Prespecified secondary endpoint	No; sample size was based on the primary efficacy endpoint	Approximately 80%	Mortality interpretation may be affected by previous ICS use and withdrawal.
KRONOS [17]	RCT; 24 weeks	Lung-function endpoints	Reported in safety follow-up	No; sample size was based on efficacy endpoints	Permitted; not restricted to participants without previous ICS use	Short follow-up and few deaths preclude mortality conclusions.
TRIBUTE [18]	RCT; 52 weeks	Moderate-to-severe exacerbation rate	Reported in safety follow-up	No; sample size was based on the primary efficacy endpoint	Variable	Previous treatment limits a pure treatment-initiation interpretation.
TRILOGY [19]	RCT; 52 weeks	FEV_1_ endpoints and exacerbation rate	Reported in safety follow-up	No; sample size was based on efficacy endpoints	Approximately 70%	ICS/LABA comparator tests adding a LAMA, not initiating ICS.
TRINITY [20]	RCT; 52 weeks	Moderate-to-severe exacerbation rate	Reported in safety follow-up	No; sample size was based on the primary efficacy endpoint	Active ICS use excluded	No contemporary LABA/LAMA comparator.
IMPACT/ETHOS re-analysis [32]	Secondary re-analysis; 2026	All-cause mortality by previous ICS use	Primary analytic focus	Not a newly powered RCT	Original trial cohorts	Constrained by subgroup data and source-trial design.
Suissa cohorts [35,36]	Observational active-comparator studies	Mortality and/or MACE	Outcome of interest	Not applicable; observational study	Adjusted when recorded	Residual confounding, channelling, and exposure misclassification remain possible.
SKOPOS-MAZI [37]	Retrospective claims study	All-cause mortality and first severe cardiopulmonary event	Co-primary outcomes	Not applicable; observational study	Balanced using IPTW	SITT-versus-MITT comparison; residual confounding and misclassification remain possible.

Note: COPD, chronic obstructive pulmonary disease; FEV_1_, forced expiratory volume in one second; ICS, inhaled corticosteroid; IPTW, inverse probability of treatment weighting; MACE, major adverse cardiovascular event; MITT, multiple-inhaler triple therapy; RCT, randomized controlled trial; SITT, single-inhaler triple therapy.

## Data Availability

No new data were created or analysed in this study. Data sharing is not applicable to this article.

## Abbreviations

The following abbreviations are used in this manuscript:

**Abbreviation**	**Definition**
BDP/FF/GB	Beclometasone dipropionate/formoterol fumarate/glycopyrronium bromide
BEC	Blood eosinophil count
BUD/GLY/FOR	Budesonide/glycopyrronium/formoterol fumarate
COPD	Chronic obstructive pulmonary disease
FEV_1_	Forced expiratory volume in one second
FF/UMEC/VI	Fluticasone furoate/umeclidinium/vilanterol
GOLD	Global Initiative for Chronic Obstructive Lung Disease
ICS	Inhaled corticosteroid
LABA	Long-acting β2-agonist
LAMA	Long-acting muscarinic antagonist
MACE	Major adverse cardiovascular event
MITT	Multiple-inhaler triple therapy
RCT	Randomized controlled trial
SITT	Single-inhaler triple therapy
TTE	Target trial emulation

## References

[1] Global Initiative for Chronic Obstructive Lung Disease (GOLD) (2026). Global Strategy for the Diagnosis, Management, and Prevention of Chronic Obstructive Pulmonary Disease: 2026 Report.

[2] Cazzola M., Bajpai J., Calzetta L., Matera M.G., Rogliani P. (2026). GOLD 2026: Transforming COPD management with early intervention, multi-dimensional assessment, and personalized care. Drugs.

[3] Csikesz N.G., Gartman E.J. (2014). New developments in the assessment of COPD: Early diagnosis is key. Int. J. Chronic Obstr. Pulm. Dis..

[4] Vogelmeier C.F., Criner G.J., Martinez F.J., Anzueto A., Barnes P.J., Bourbeau J., Celli B.R., Chen R., Decramer M., Fabbri L.M. (2017). Global strategy for the diagnosis, management, and prevention of chronic obstructive lung disease 2017 report: GOLD executive summary. Eur. Respir. J..

[5] Singh D., Agusti A., Martinez F.J., Papi A., Pavord I.D., Wedzicha J.A., Vogelmeier C.F., Halpin D.M.G. (2022). Blood Eosinophils and Chronic Obstructive Pulmonary Disease: A Global Initiative for Chronic Obstructive Lung Disease Science Committee 2022 Review. Am. J. Respir. Crit. Care Med..

[6] Alsuwaigh R., Yii A., Loh C.H., Xu X., Bahety P., Navarro Rojas A.A., Milea D., Tee A. (2024). A comparison of GOLD 2019 and 2023 recommendations to contemporaneous real-world inhaler treatment patterns for chronic obstructive pulmonary disease management in Singapore. J. Thorac. Dis..

[7] Chalmers J.D., Laska I.F., Franssen F.M.E., Janssens W., Pavord I., Rigau D., McDonnell M.J., Roche N., Sin D.D., Stolz D. (2020). Withdrawal of inhaled corticosteroids in COPD: A European Respiratory Society guideline. Eur. Respir. J..

[8] Sun Y., Zhou J. (2019). New insights into early intervention of chronic obstructive pulmonary disease with mild airflow limitation. Int. J. Chronic Obstr. Pulm. Dis..

[9] Vanfleteren L.E.G.W., Fabbri L.M., Papi A., Petruzzelli S., Celli B.R. (2018). Triple therapy (ICS/LABA/LAMA) in COPD: Time for a reappraisal. Int. J. Chronic Obstr. Pulm. Dis..

[10] Calzetta L., Rogliani P., Matera M.G., Cazzola M. (2016). A systematic review with meta-analysis of dual bronchodilation with LAMA/LABA for the treatment of stable COPD. Chest.

[11] Ismaila A.S., Huisman E.L., Punekar Y.S., Karabis A. (2015). Comparative efficacy of long-acting muscarinic antagonist monotherapies in COPD: A systematic review and network meta-analysis. Int. J. Chronic Obstr. Pulm. Dis..

[12] Hernán M.A., Wang W., Leaf D.E. (2022). Target trial emulation: A framework for causal inference from observational data. JAMA.

[13] Wing K., Leyrat C. (2025). Improving the validity of noninterventional comparative effectiveness research by basing study design on a specified existing randomized controlled trial. Am. J. Epidemiol..

[14] Suissa S., Schneeweiss S., Feldman W.B., Tesfaye H., Wang S.V. (2025). Emulating randomized trials by observational database studies: The RCT-DUPLICATE initiative in COPD and asthma. Am. J. Epidemiol..

[15] Lipson D.A., Barnhart F., Brealey N., Brooks J., Criner G.J., Day N.C., Dransfield M.T., Halpin D.M.G., Han M.K., Jones C.E. (2018). Once-daily single-inhaler triple versus dual therapy in patients with COPD. N. Engl. J. Med..

[16] Rabe K.F., Martinez F.J., Ferguson G.T., Wang C., Singh D., Wedzicha J.A., Trivedi R., St Rose E., Ballal S., McLaren J. (2020). Triple inhaled therapy at two glucocorticoid doses in moderate-to-very-severe COPD. N. Engl. J. Med..

[17] Ferguson G.T., Rabe K.F., Martinez F.J., Fabbri L.M., Wang C., Ichinose M., Bourne E., Ballal S., Darken P., DeAngelis K. (2018). Triple therapy with budesonide/glycopyrrolate/formoterol fumarate with co-suspension delivery technology versus dual therapies in chronic obstructive pulmonary disease (KRONOS): A double-blind, parallel-group, multicentre, phase 3 randomised controlled trial. Lancet Respir. Med..

[18] Papi A., Vestbo J., Fabbri L., Corradi M., Prunier H., Cohuet G., Guasconi A., Montagna I., Vezzoli S., Petruzzelli S. (2018). Extrafine inhaled triple therapy versus dual bronchodilator therapy in chronic obstructive pulmonary disease (TRIBUTE): A double-blind, parallel-group, randomised controlled trial. Lancet.

[19] Singh D., Papi A., Corradi M., Pavlišová I., Montagna I., Francisco C., Cohuet G., Vezzoli S., Scuri M., Vestbo J. (2016). Single inhaler triple therapy versus inhaled corticosteroid plus long-acting β_2_-agonist therapy for chronic obstructive pulmonary disease (TRILOGY): A double-blind, parallel-group, randomised controlled trial. Lancet.

[20] Vestbo J., Papi A., Corradi M., Blazhko V., Montagna I., Francisco C., Cohuet G., Vezzoli S., Scuri M., Singh D. (2017). Single inhaler extrafine triple therapy versus long-acting muscarinic antagonist therapy for chronic obstructive pulmonary disease (TRINITY): A double-blind, parallel-group, randomised controlled trial. Lancet.

[21] Lai C.C., Chen C.H., Lin C.Y.H., Wang C.Y., Wang Y.H. (2019). The effects of single inhaler triple therapy vs. single inhaler dual therapy or separate triple therapy for the management of chronic obstructive pulmonary disease: A systematic review and meta-analysis of randomized controlled trials. Int. J. Chronic Obstr. Pulm. Dis..

[22] Rothnie K.J., Wood R.P., Czira A., Banks V.L., Camidge L.J., Massey O.K.I., Seif M., Compton C., Sharma R., Halpin D.M.G. (2024). Outcomes of patients with COPD switching from multiple-inhaler to once-daily single-inhaler triple therapy in a real-world primary care setting in England: A retrospective pre–post cohort study. BMJ Open Respir. Res..

[23] Cazzola M., Rogliani P., Calzetta L., Matera M.G. (2018). Triple therapy versus single and dual long-acting bronchodilator therapy in COPD: A systematic review and meta-analysis. Eur. Respir. J..

[24] Zheng Y., Zhu J., Liu Y., Lai W., Lin C., Qiu K., Wu J., Yao W. (2018). Triple therapy in the management of chronic obstructive pulmonary disease: Systematic review and meta-analysis. BMJ.

[25] Brusselle G., Bakakos P., Stolz D., Piraino A., Franzini L., Nudo E., Di Palma L., Rogliani P. (2026). BDP/FF/GB single-inhaler triple therapy in COPD: Real-world effectiveness, safety, and adherence in a pooled analysis of 5000 patients. Respir. Med..

[26] Lim H., Kim D.H., Hong S.H., Shin J., Lee H., Shin K., Ra S.W., Lee S.J. (2025). Real-world effectiveness of single-inhaler triple treatment through assorted respiratory outcomes when switched from multiple-inhaler triple therapies (RESTART): A prospective cohort study of Korean patients with COPD. Int. J. Chronic Obstr. Pulm. Dis..

[27] Halpin D.M.G., Worsley S., Ismaila A.S., Beeh K.M., Midwinter D., Kocks J.W.H., Irving E., Marin J.M., Martin N., Tabberer M. (2021). INTREPID: Single- versus multiple-inhaler triple therapy for COPD in usual clinical practice. ERJ Open Res..

[28] Calverley P.M.A., Anderson J.A., Celli B., Ferguson G.T., Jenkins C., Jones P.W., Yates J.C., Vestbo J. (2007). Salmeterol and fluticasone propionate and survival in chronic obstructive pulmonary disease. N. Engl. J. Med..

[29] Lipson D.A., Crim C., Criner G.J., Day N.C., Dransfield M.T., Halpin D.M.G., Han M.K., Jones C.E., Kilbride S., Lange P. (2020). Reduction in all-cause mortality with fluticasone furoate/umeclidinium/vilanterol in patients with chronic obstructive pulmonary disease. Am. J. Respir. Crit. Care Med..

[30] Suissa S., Drazen J.M. (2018). Making sense of triple inhaled therapy for COPD. N. Engl. J. Med..

[31] Soumagne T., Zysman M., Karadogan D., Lahousse L., Mathioudakis A.G. (2023). Impact of triple therapy on mortality in COPD. Breathe.

[32] Leung K., Hingorani A.D. (2026). Inhaled corticosteroid withdrawal and mortality rate in trials of triple therapy inhalers for COPD. Thorax.

[33] Ioannides A.E., Quint J.K. (2026). Is it time to re-evaluate the levels of evidence in medical research: Commentary on inhaled corticosteroids in COPD. Thorax.

[34] Wedzicha J.A., Banerji D., Chapman K.R., Vestbo J., Roche N., Ayers R.T., Thach C., Fogel R., Patalano F., Vogelmeier C.F. (2016). Indacaterol–glycopyrronium versus salmeterol–fluticasone for COPD. N. Engl. J. Med..

[35] Suissa S., Dell’Aniello S., Ernst P. (2025). Single-inhaler triple vs. long-acting β_2_-agonist–inhaled corticosteroid therapy for COPD: Comparative safety in real-world clinical practice. Chest.

[36] Suissa S., Dell’Aniello S., Ernst P. (2022). Triple inhaler versus dual bronchodilator therapy in COPD: Real-world effectiveness on mortality. J. Chronic Obstr. Pulm. Dis..

[37] Pollack M., Rapsomaniki E., Anzueto A., Rhodes K., Hawkins N.M., Vogelmeier C.F., Marshall J., Müllerová H. (2025). Effectiveness of single versus multiple inhaler triple therapy on mortality and cardiopulmonary risk reduction in COPD: The SKOPOS-MAZI study. Am. J. Med..

[38] Hammadi A., Hoyas-Sánchez C., Romero-Linares A., Álvarez-Muro L., Menéndez-Lobo A., Romeral-Navarro D., Jiménez-Antón A., Almansa-López A., Casares-Martín-Moreno L., Sánchez-Álvarez E. (2025). All-cause and cardiovascular mortality with single inhaler triple therapy versus double therapies for COPD: A systematic review and metanalysis. Arch. Bronconeumol..

[39] Kew K.M., Seniukovich A. (2014). Inhaled steroids and risk of pneumonia for chronic obstructive pulmonary disease. Cochrane Database Syst. Rev..

[40] Mkorombindo T., Dransfield M.T. (2020). Inhaled corticosteroids in chronic obstructive pulmonary disease: Benefits and risks. Clin. Chest Med..

[41] Brusselle G., Pavord I.D., Landis S., Pascoe S., Lettis S., Morjaria N., Barnes N., Hilton E. (2018). Blood eosinophil levels as a biomarker in COPD. Respir. Med..

[42] Watz H., Tetzlaff K., Wouters E.F.M., Kirsten A., Magnussen H., Rodriguez-Roisin R., Vogelmeier C., Fabbri L.M., Chanez P., Dahl R. (2016). Blood eosinophil count and exacerbations in severe chronic obstructive pulmonary disease after withdrawal of inhaled corticosteroids: A post-hoc analysis of the WISDOM trial. Lancet Respir. Med..

[43] Chan M.C., Yeung Y.C., Yu E.L.M., Yu W.C. (2020). Blood eosinophil and risk of exacerbation in chronic obstructive pulmonary disease patients: A retrospective cohort analysis. Int. J. Chronic Obstr. Pulm. Dis..

[44] Bafadhel M., Davies L., Calverley P.M.A., Aaron S.D., Brightling C.E., Pavord I.D. (2014). Blood eosinophil guided prednisolone therapy for exacerbations of COPD: A further analysis. Eur. Respir. J..

[45] Casanova C., Celli B.R., de-Torres J.P., Martínez-Gonzalez C., Cosio B.G., Pinto-Plata V., de Lucas-Ramos P., Divo M., Fuster A., Peces-Barba G. (2017). Prevalence of persistent blood eosinophilia: Relation to outcomes in patients with COPD. Eur. Respir. J..

[46] Suissa S., Dell’Aniello S., Ernst P. (2018). Comparative effectiveness of LABA–ICS versus LAMA as initial treatment in COPD targeted by blood eosinophils: A population-based cohort study. Lancet Respir. Med..

[47] Dahl R., Jadayel D., Alagappan V.K., Chen H., Banerji D. (2013). Efficacy and safety of QVA149 compared to the concurrent administration of its monocomponents indacaterol and glycopyrronium: The BEACON study. Int. J. Chronic Obstr. Pulm. Dis..

[48] Chapman K.R., Hurst J.R., Frent S.M., Larbig M., Fogel R., Guerin T., Banerji D., Patalano F., Goyal P., Pfister P. (2018). Long-term triple therapy de-escalation to indacaterol/glycopyrronium in patients with chronic obstructive pulmonary disease (SUNSET): A randomized, double-blind, triple-dummy clinical trial. Am. J. Respir. Crit. Care Med..

[49] Magnussen H., Disse B., Rodriguez-Roisin R., Kirsten A., Watz H., Tetzlaff K., Towse L., Finnigan H., Dahl R., Decramer M. (2014). Withdrawal of inhaled glucocorticoids and exacerbations of COPD. N. Engl. J. Med..

[50] Suissa S., Coulombe J., Ernst P. (2015). Discontinuation of inhaled corticosteroids in COPD and the risk reduction of pneumonia. Chest.

[51] Sposato B., Lacerenza L.G., Croce S., Petrucci E., Fabbrini V., Giannini L., Baratta P., Cresti A., Ricci A., Micheletto C. (2025). Real-life efficacy of single-inhaler triple therapy with budesonide/glycopyrronium/formoterol fumarate in persistent COPD users: A retrospective database study. Biomedicines.

[52] Young C., Lee L.Y., DiRocco K.K., Germain G., Klimek J., Laliberté F., Lejeune D., Noorduyn S.G., Paczkowski R. (2025). Adherence and persistence with single-inhaler triple therapy among patients with COPD using commercial and Medicare Advantage US health plan claims data. Adv. Ther..

[53] Paczkowski R., Sharma R., Sreedharan S., Payne P., Tritton T., Claussen J., Noorduyn S.G. (2024). Comparing patient adherence and persistence to single-inhaler triple therapy vs. multiple-inhaler triple therapy: A narrative review of real-world observational studies. Chest.

[54] González-González M.A., Pedrosa-Naudín M.A., Fernández-Lázaro D., Planelles I.D., Álvarez F.J., Gutiérrez-Abejón E. (2025). Single- versus multiple-inhaler triple therapy in patients with COPD in Spain: A retrospective cohort study comparing adherence, persistence, risk of exacerbations and economic outcomes. Front. Pharmacol..

[55] Requena G., Camidge L.J., Ford A., Yarita M., Hashimoto K., Jennison T., Massey O.S., Noorduyn S.G., Mizukami A. (2025). Effectiveness of switching from multiple-inhaler to once-daily single-inhaler triple therapy in patients with COPD in a real-world setting in Japan. Int. J. Chronic Obstr. Pulm. Dis..

[56] Mäkelä M.J., Backer V., Hedegaard M., Larsson K. (2013). Adherence to inhaled therapies, health outcomes and costs in patients with asthma and COPD. Respir. Med..

[57] Rogliani P., Ora J., Puxeddu E., Matera M.G., Cazzola M. (2017). Adherence to COPD treatment: Myth and reality. Respir. Med..

[58] van der Palen J., Thomas M., Chrystyn H., Sharma R.K., van der Valk P.D., Goosens M., Wilkinson T., Stonham C., Chauhan A.J., Imber V. (2016). A randomised open-label cross-over study of inhaler errors, preference and time to achieve correct inhaler use in patients with COPD or asthma: Comparison of ELLIPTA with other inhaler devices. npj Prim. Care Respir. Med..

[59] Chrystyn H., van der Palen J., Sharma R., Barnes N., Delafont B., Mahajan A., Thomas M. (2017). Device errors in asthma and COPD: Systematic literature review and meta-analysis. npj Prim. Care Respir. Med..

[60] Toy E.L., Beaulieu N.U., McHale J.M., Welland T.R., Plauschinat C.A., Swensen A., Duh M.S. (2011). Treatment of COPD: Relationships between daily dosing frequency, adherence, resource use, and costs. Respir. Med..

[61] Price D., Brusselle G., Gruffydd-Jones K., Miravitlles M., Stewart R., Baldwin M., Keininger D.L., Jones R.C. (2015). The inevitable drift to triple therapy in COPD: An analysis of prescribing pathways in the UK. Int. J. Chronic Obstr. Pulm. Dis..

[62] Monteagudo M., Barrecheguren M., Solntseva I., Dhalwani N., Booth A., Nuñez A., Lambrelli D., Miravitlles M. (2021). Clinical characteristics and factors associated with triple therapy use in newly diagnosed patients with COPD. npj Prim. Care Respir. Med..

[63] Agenzia Italiana del Farmaco (AIFA) Nota 99: Medicinali per uso Inalatorio per la Terapia di Mantenimento della BPCO. https://www.aifa.gov.it/nota-99.

[64] Canadian Agency for Drugs and Technologies in Health (CADTH) (2020). Coverage Categories at Public Drugs Plans in Canada: Environmental Scan.

[65] Fenwick E., Martin A., Schroeder M., Mealing S.J., Solanke O., Risebrough N., Ismaila A.S. (2021). Cost-effectiveness analysis of a single-inhaler triple therapy for COPD in the UK. ERJ Open Res..

[66] Beeh K.M., Claussen J., Shah D., Martin A., Kendall R., Dasari P., Ismaila A.S., Noorduyn S.G. (2025). Cost-effectiveness of single-inhaler versus multiple-inhaler triple therapy in COPD: A German healthcare perspective. Int. J. Chronic Obstr. Pulm. Dis..

[67] Rana R.H., Alam K., Keramat S.A., Gow J. (2022). Cost-effectiveness of single-inhaler triple therapy for patients with severe COPD: A systematic literature review. Expert Rev. Respir. Med..

[68] Ismaila A.S., Risebrough N., Schroeder M., Shah D., Martin A., Goodall E.C., Ndirangu K., Criner G.J., Dransfield M.T., Halpin D.M.G. (2019). Cost-effectiveness of once-daily single-inhaler triple therapy in COPD: The IMPACT trial. Int. J. Chronic Obstr. Pulm. Dis..

[69] Punekar Y.S., Shukla A., Müllerová H. (2014). COPD management costs according to the frequency of COPD exacerbations in UK primary care. Int. J. Chronic Obstr. Pulm. Dis..

[70] Dilokthornsakul P., Dilokthornsakul P., Chongmelaxme B., Niyompattama A. (2026). Economic evaluation of single-inhaler triple therapy for chronic obstructive pulmonary disease in Thailand. BMJ Open Respir. Res..

[71] Dimic-Janjic S., Stjepanovic M., Cekerevac I., Hromis S., Buha I., Cupurdija V., Kopitovic I., Milic R., Zvezdin B., Stankovic I. (2026). Discharge practices after hospitalization for COPD exacerbations: A physician survey and SWOT analysis. Healthcare.

